# Radiological risk assessment of natural radioactivity in imported rice consumed in Ghana and its implications for food safety and public health

**DOI:** 10.1038/s41598-026-37317-0

**Published:** 2026-01-27

**Authors:** Philomena Dickson-Agudey, Lordford Tettey-Larbi, Serwaa Adjei-Kyereme, Henry Lawluvi, Eric Ofosu Asare, Rita Kwabea Osei, Andriana Asare Ampene, Amin Shahrokhi

**Affiliations:** 1 Nuclear Regulatory Authority, Kwabenya, Ghana; 2 School of Nuclear and Allied Sciences, University of Ghana, Accra, Ghana; 3 Department of Radiochemistry and Radioecology, Research Centre for Biochemical, Environmental and Chemical Engineering, University of Pannonia, Veszprém, Hungary; 4 Radiation Protection Institute, Ghana Atomic Energy Commission, Accra, Ghana

**Keywords:** Imported food safety, Regulatory framework, Radiation, ingestion exposure, HPGe detector, Public health standards, Environmental sciences, Environmental social sciences, Risk factors

## Abstract

Rice is a staple food in many developing countries, and its role in public health has been a focus of research for decades. In Ghana, a developing country in sub-Saharan Africa, per capita rice consumption is estimated at 50 kg per person per year (2023/2024 marketing year). Meanwhile, in the same period, Ghana’s milled rice production was estimated at 1.009 million metric tons, covering about 59% of the national consumption needs. And this is while, many developing countries often lack well-defined regulatory frameworks for pre-import evaluation of critical safety parameters in food products. To bridge the consumption gap, Ghana imports a significant portion of its rice, with imports forecasted at 950,000 metric tons. Despite the significance of imported rice in the national food supply, there is a notable lack of comprehensive radiological assessments focused on these products. Therefore, this study attempts to address this gap by analyzing radioactive contaminants—radium-226, radium-228, and potassium-40 —in imported rice consumed in Ghana. A total of 50 rice samples, with an average of 10 samples per brand from 5 distinct brands were collected from major markets in Accra, Kumasi, Tamale and analyzed using gamma-ray spectrometry with a High Purity Germanium (HPGe) detector. The mean activity concentrations were 2.13 ± 0.85 Bq/kg for ^226^Ra, 3.69 ± 1.47 Bq/kg for ^228^Ra, and 51.82 ± 7.93 Bq/kg for ^40^K, all within international safety limits. The estimated total annual effective dose for an average adult was 408.44 µSv/y well below recommended international thresholds. The excess lifetime cancer risk (ELCR) was also calculated, with results indicating a negligible impact on public health. These findings highlight the importance of continuous monitoring of radioactive contaminants in imported food products to ensure consumer safety. Additionally, the study provides critical insights for policymakers, emphasizing the need for regulatory measures to control radioactivity levels in imported foods. This research supports Ghana’s public health initiatives and alignment with global food safety standards.

## Introduction

The ingestion of natural radionuclides through food has become an increasing public health concern due to its potential long-term radiological risks. Rice (*Oryza sativa*), a staple food for a significant portion of the global population, serves as a major pathway for radionuclide intake in humans. Research has shown that rice can uptake trace to accumulated quantities of natural radioactivity, primarily from radionuclides such as radium-226 (^226^Ra), radium-228 (^228^Ra), and potassium-40 (^40^K) during photosynthesis through root uptake of soluble radionuclides in soil water, atmospheric deposition and soil resuspension^1,2^. While these radionuclides are naturally present in the environment, their accumulation in food products, especially rice, raises concerns about prolonged exposure and its potential health implications especially with their alpha emitting properties^3^.

In regions where rice is a dietary staple, assessing its natural radioactivity and associated risks is critical for public health. For example, a study in Bangladesh reported average activity concentrations of 1.09 Bq/kg for ^226^Ra, 0.17 Bq/kg for ^228^Ra, and 4.70 Bq/kg for ^40^K in rice samples^4^. The estimated annual effective dose for adult consumers was approximately 64.23 µSv below international safety limits but still warranting continuous monitoring due to the cumulative nature of radiation exposure^4^. These findings emphasize the need for similar evaluations in other regions, particularly in countries that rely on imported rice.

Ghana, like many West African nations, depends heavily on imported rice from India, Malaysia, Vietnam, Thailand, China, Pakistan, United States of America as well as Cote d’Ivoire and Nigeria to meet domestic demand^5^. The radiological quality of imported rice is influenced by environmental conditions in its country of origin, which vary significantly based on factors such as soil composition, agricultural practices, and geological characteristics^6^. Differences in natural radioactivity levels across rice-producing regions could expose consumers to varying degrees of radiological risk. For this reason, assessing the radiological content of imported rice is essential for public health safety and alignment with global food safety standards.

Despite growing global attention on radiation exposure through food, limited research has focused on the radiological health risks of imported rice in Ghana. This study aims to address this gap by analysing radioactive contaminants in imported rice consumed in Ghana and estimating the associated health risks. The findings will contribute valuable data to inform public health policies, enhance consumer safety, and establish a framework for ongoing monitoring and regulation of radioactive contaminants in imported food products.

This research is particularly relevant considering the increasing international efforts to strengthen food safety standards and improve radiological protection measures. This study aligns with international guidelines and would contribute to the global discourse on food safety.

## Materials and methods

To assess radiological health risks associated with imported rice consumption in Ghana, ten (10) samples per five (5) common brands of rice samples were collected. The selected rice varieties represent the most consumed imported brands available in the Ghanaian market.

Following established radiological study protocols, each sample was placed in an airtight plastic bag to prevent contamination, labelled with a unique identifier and collection date for accurate record-keeping, and transported to the Radiation Monitoring Laboratory at the Ghana Atomic Energy Commission for analysis^4^.

Upon arrival at the laboratory, the samples were ground into a fine powder using a mortar and pestle and sieved through a 400 μm mesh to achieve homogeneity and for consistent packing density and detector geometry^4^. The sieved samples were then placed into standardized cylindrical containers, sealed, and stored for a minimum of 30 days to establish secular equilibrium between radium and thorium progeny^7^.

### Sample measurement

Gamma-ray spectrometry was used to determine the activity concentrations of radionuclides ^226^Ra, ^228^Ra, and ^40^K in the rice samples. A High Purity Germanium (HPGe) detector with a relative efficiency of 20% and an energy resolution of 1.8 keV-FWHM at 1332.5 keV was employed, following the methodology outlined by Nahar et al.^4^.

To ensure measurement accuracy, the system was calibrated using standard gamma-ray sources ^60^Co, ^137^Cs, and ^152^Eu from the International Atomic Energy Agency (IAEA)^8^. Each rice sample was counted for approximately 80,000 s, and net counts were obtained by subtracting background radiation levels from total recorded counts. The activities of ^226^Ra and ^228^Ra were indirectly determined via their daughter products ^214^Pb, ^214^Bi, ^212^Pb, ^208^Tl, and ^228^Ac under the assumption of secular equilibrium after the 30 days waiting period^8^.

#### Measurement uncertainties

In order to ensure the accuracy of the estimated activity concentrations, total measurement uncertainties were assessed by accounting for contributions from counting statistics, energy and efficiency calibration, detector background, and peak fitting. Counting statistical uncertainties were derived from the square root of the net peak area and propagated through the activity concentration equation. Efficiency calibration uncertainties (typically 3–5%) were obtained from the fitted efficiency curve constructed using IAEA-certified standard sources (^60^Co, ^137^Cs, and ^152^Eu). Energy calibration uncertainties were small (< 0.2%) but included in the propagation. These individual components were combined using standard uncertainty-propagation procedures, and all reported activity concentrations represent arithmetic mean values ± total propagated uncertainty. These uncertainties define the confidence intervals of the reported results but do not affect sample-to-sample comparison since all samples were analyzed under identical conditions.

### Activity concenctration calculation

The activity concentration A_C_ (Bq/kg) of each radionuclide was calculated using the equation:

1 $$\:{A}_{C}=\frac{{N}_{D}}{P.{T}_{c}.\eta\:\left(E\right).M}$$

where:

- N_D_ = net counts.
- P = gamma-ray emission probability.
- T_c_ = counting time (80,000 s).
- η(E) = absolute counting efficiency of the detector.
- M = sample weight (kg).

This approach ensures accurate quantification of radionuclide concentrations^9^.

### Minimum detectable activity

The Minimum Detectable Activity (MDA) of the HPGe detector system was determined using Currie’s (1968) equation^10^:

2 $$\:MDA=\frac{2.71+4.65\surd\:B}{{\upepsilon\:}{\upgamma\:}\times\:\mathrm{I}{\upgamma\:}\times\:\mathrm{T}}$$

​where:

- B = background counts in the region of interest.
- εγ = detector efficiency at the respective gamma-ray energy.
- Iγ = gamma-ray emission probability.
- T = counting time (s).

The Minimum detectable activity (MDA) for the radionuclides of interest for this study were estimated to be 0.983 Bq for ^214^Pb, 0.6644 Bq for ^214^Bi at 609.3 keV, 0.2355 Bq for ^214^Bi at 1120.3 keV, 0.464 Bq for ^212^Pb at 238.6 keV, 0.689 Bq for ^208^Tl at 583.2 keV, 1.124 Bq for ^228^Ac at 911.1 keV, and 0.549 Bq for ^40^K at 1460.83 keV.

### Annual committed effective dose

The annual committed effective dose E_eff_ (mSv/year) due to radionuclide intake was estimated as:

3 $$\:{E}_{eff}={A}_{C}\times\:{A}_{ig}\times\:{D}_{cf}$$

where:

- A_C_ = average activity concentration of radionuclides (Bq/kg).
- A_ig_ = annual per capita rice consumption in Ghana (50 kg/year)^5^.
- D_cf._= ingestion dose conversion factor for the radionuclides of interest; for ^226^Ra, ^228^Ra, and ^40^K to be 2.8 × 10^− 7^ Sv/Bq, 6.9 × 10^− 7^ Sv/Bq, and 6.2 × 10^− 9^ Sv/Bq respectively^11,12^.

This study assumed secular equilibrium between ^226^Ra and ^228^Ra and their short-lived progeny after the 30-day sealing period for dose estimations. It also assumed homogeneous radionuclide dispersion in the rice matrix and uniform adult Ghanaian rice consumption rate. Minor deviations from secular equilibrium or consumption rates may contribute minor uncertainty, although these assumptions meet IAEA protocols and are routinely used in food radioactivity investigations.

### Excess lifetime cancer risk

To assess long-term health risks, the Excess Lifetime Cancer Risk (ELCR) was calculated using:

4 $$\:ELCR={E}_{eff}\times\:LS\times\:{R}_{c}$$

where:

- E_eff_ = Annual Committed Effective Dose.
- LS = average lifespan (assumed to be 70 years).
- R_c_ = ingestion risk coefficient (0.05 per Sv)^11,12^.

## Results and discussion

### Activity concentrations of radionuclides

From Table 1, the mean measured activity concentrations in imported rice samples per brand across the 10 samples were 2.13 ± 0.85 Bq/kg for ^226^Ra, 3.69 ± 1.47 Bq/kg for ^228^Ra, and 51.82 ± 7.93 Bq/kg for ^40^K. These values are within the international safety limits recommended by UNSCEAR^11^.

**Table 1:** Activity concentrations of radionuclides in imported rice [(Bq/kg) dry weight]

Rice Sample	^226^Ra	^228^Ra	^40^K
RS1	2.12 ± 1.84	4.85 ± 1.75	78.30 ± 13.84
RS2	1.09 ± 0.24	3.17 ± 1.81	26.10 ± 2.52
RS3	3.04 ± 1.31	1.98 ± 0.34	54.12 ± 8.95
RS4	1.80 ± 0.56	4.10 ± 1.81	51.47 ± 8.59
RS5	1.61 ± 0.48	4.35 ± 1.63	47.12 ± 6.74
**Average**	**2.13 ± 0.85**	**3.69 ± 1.47**	**51.82 ± 7.93**

**Fig. 1:**
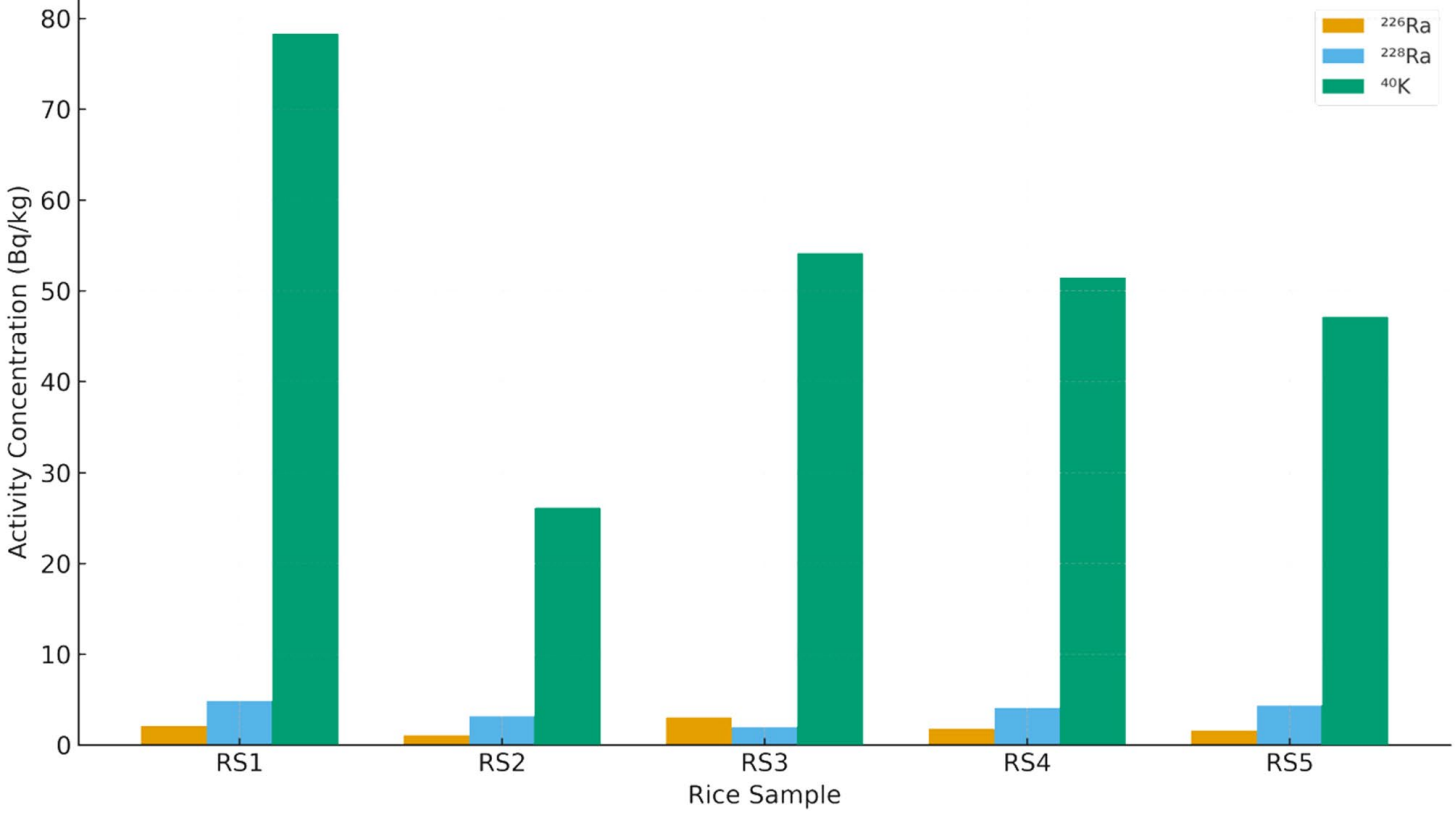
– Activity concentration distribution ^226^Ra, ^228^Ra, and ^40^K across all sample group.

As observed in Fig. 1, the high relative values of ^228^Ra than ^226^Ra may be due to ^232^Th enrich soil farms on which these rices were grown, while potassium values are expected because of its abundance in environmental media including foodstuff.

The results indicate slight variations compared to studies conducted in other rice-consuming regions (Table 2).

**Table 2:** Comparison of radionuclide concentrations (Bq/kg) dry weight in rice across different Countries.

Country	^226^Ra	^228^Ra	^40^K	Source
Bangladesh	0.47–1.66	0.04–0.49	1.09–9.23	^4^
	1.00–30.10	2.80–36.00	10.60–128.80	^13^
India	0.01 – 0.30	0.08–0.56	45.90–86.60	^14^
Iran	0.02–0.67	0.03–0.20	7.10–111.00	^15^
	1.27–2.89	0.42–15.24	84.66–122.66	^16^
Malaysia	18.33–25.10	35.49–64.97	64.80–109.93	^17^
	1.50–2.80	3.60–7.50	59.90–92.20	^7^
	56.97–86.13	34.71–52.14	517.05–997.59	^18^
Nigeria	5.73–13.46	5.90–13.20	35.96–87.89	^19^
	0.90–2.40	0.50–2.40	78.00–111.00	^20^
Saudi Arabia	0.10–2.60	0.10–2.30	45.00–257.20	^21^
Kuwait	0.41–0.91	0.32–0.62	32.90–101.00	^22^
Ghana	2.61–6.92*	3.61–9.50*	41.70–210.80*	^23^
	1.09–3.04	1.98–4.85	26.10–78.30	[Present study]

* Locally grown rice.

As shown in Table 2, the analysis revealed that although the activity concentrations of ^226^Ra, ^228^Ra, and ^40^K in the imported rice samples in Ghana compares well with other studies in other regions, there were in some cases, significant differences; even compared with previous studies on locally grow rice^4,7,13–23^. The variations arise from differences in soil composition, agricultural practices, and environmental factors. However, a statistical independent-sample t-test (*p* < 0.05) confirmed that the variations between our findings and those of other studies were not significant. This suggests that the observed differences fall within expected global fluctuations in natural radionuclide levels in rice. Nonetheless, the results were consistent with those studies’ levels and remained within internationally range levels for grains which include rice with 0.7 mBq/kg to 5.2 Bq/kg for ^226^Ra, 0.04 mBq/kg to 6.2 Bq/kg for ^228^Ra and no reported reference values for ^40^K^11^. There is no internationally reported reference value of ingested ^40^K as it is considered to be of no or insignificant internal exposure radiologically risk and the human body also has its metabolism of removing excess potassium (including ^40^K) from the body^11^.

### Radiological risk parameters

#### Annual effective dose

The annual effective dose (AED) for ^226^Ra, ^228^Ra, and ^40^K due to rice consumption was calculated using dose conversion factors from^11,12^. The results are summarized in Table 3. The estimated annual effective dose associated with the consumption of imported rice in Ghana also remained below the safety limits while aligning with similar studies from Malaysia, Bangladesh, India, Iran, Nigeria, Saudi Arabia, Kuwait, and Greece^7,11,13–22,24,25^.

**Table 3:** Annual effective dose (µSv/year) from radionuclides in rice Samples.

Rice Sample	^226^Ra	^228^Ra	^40^ K	Total
RS1	84.45	335.28	69.09	488.82
RS2	43.39	219.01	23.03	285.43
RS3	121.22	136.71	47.80	305.73
RS4	71.75	282.94	45.47	400.16
RS5	64.20	300.52	41.53	406.09
**Average**	**76.99**	**254.89**	**45.38**	**377.25**

The variabilities in the estimated annual effective dose as show in Table 3 arise primarily from the propagated variabilities in activity concentrations, variability in annual rice consumption rates across individuals, and inherent uncertainties in ingestion dose conversion factors, which are estimated by the ICRP to vary by ± 20–30%. Despite these uncertainties, the resulting variability does not alter the overall conclusion that the doses are well below international reference levels (1 mSv/year) and hence poses negligible radiological risk for adult consumers.

#### Excess lifetime cancer risk

The excess lifetime cancer risk (ELCR) was calculated based on the estimated annual effective dose of 0.05 Sv^−1^ for the public (Table 4). The results indicated an average ELCR value of 0.76 × 10^−3^, which is less than the world average value of 1.165 × 10^−3^ recommended by for internal exposure^11,12^.

**Table 4:** Excess lifetime cancer risk from radionuclides in rice samples.

Rice Sample	Ra-226	Ra-228	K-40	Total
RS1	3.22 × 10^−4^	7.38 × 10^−4^	2.45 × 10^−5^	1.07 × 10^−3^
RS2	1.65 × 10^−4^	4.82 × 10^−4^	8.17 × 10^−5^	0.65 × 10^−3^
RS3	4.61 × 10^−4^	3.00 × 10^−4^	1.69 × 10^−5^	0.78 × 10^−3^
RS4	2.73 × 10^−4^	6.22 × 10^−4^	1.61 × 10^−5^	0.61 × 10^−3^
RS5	2.44 × 10^−4^	6.60 × 10^−4^	1.47 × 10^−5^	0.69 × 10^−3^
Average	2.93 × 10^−4^	5.60 × 10^−4^	1.61 × 10^−5^	0.76 × 10^−3^

To put this into context, the estimated ELCR from rice consumption is comparable to values reported in other regions with high rice dependency, such as Bangladesh, India, Iran, Malaysia, and Nigeria^4,7,13–20^. The risk remains low for the general adult population; however, due to differences in metabolic rates and dietary intake, children and pregnant women may be more susceptible to radiation exposure effects^8,11^.

**Fig. 2:**
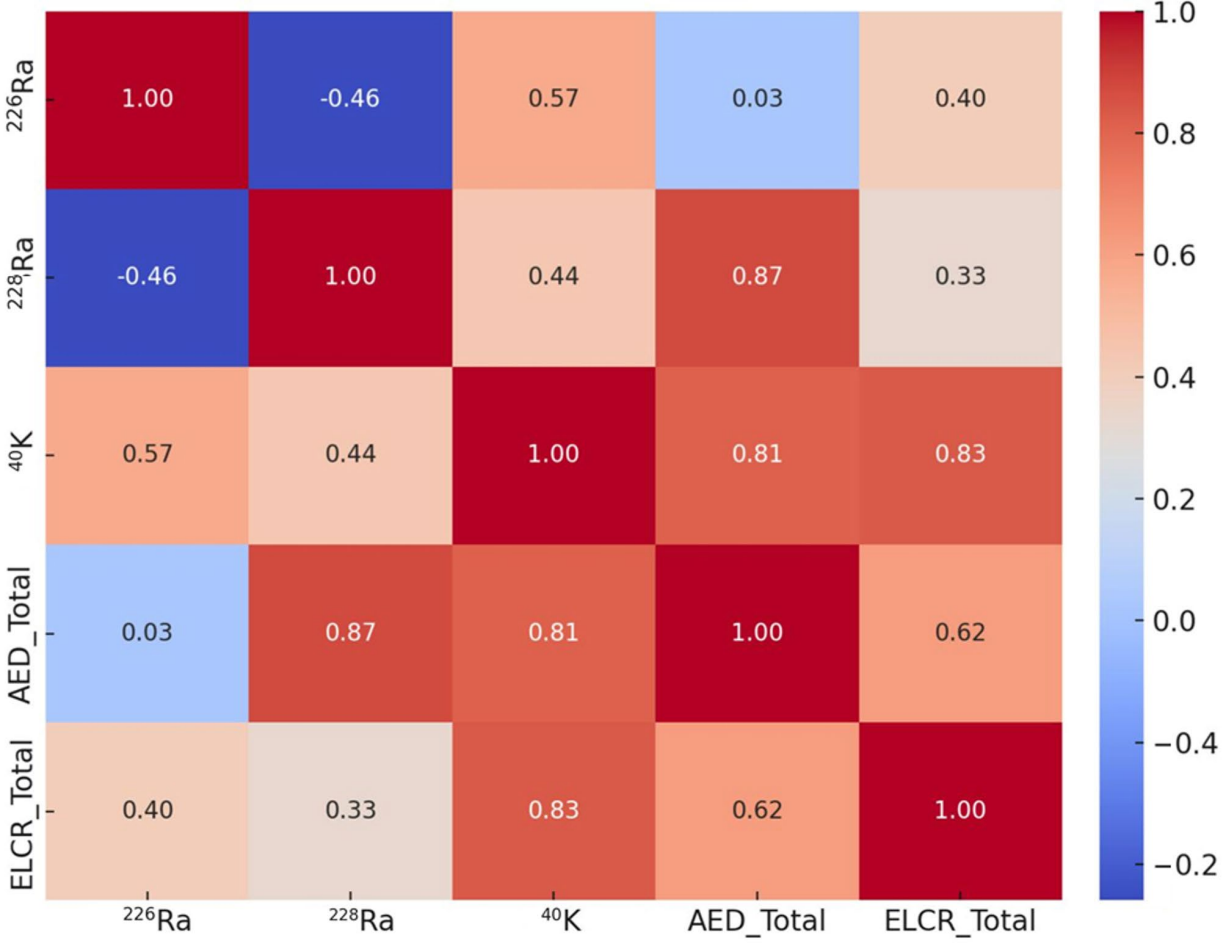
– Correlation matrix for radionuclide concentrations and associated radiological parameters.

Figure 2 shows a deeper understanding of the relationship between radionuclide concentrations and associated radiological risks. Strong positive correlations between the radionuclides and radiological risk parameters, indicating that increased concentrations of radionuclides relate to higher radiological risks. Particularly strong relationships between ^228^Ra concentrations and annual effective dose (AED).

**Fig. 3:**
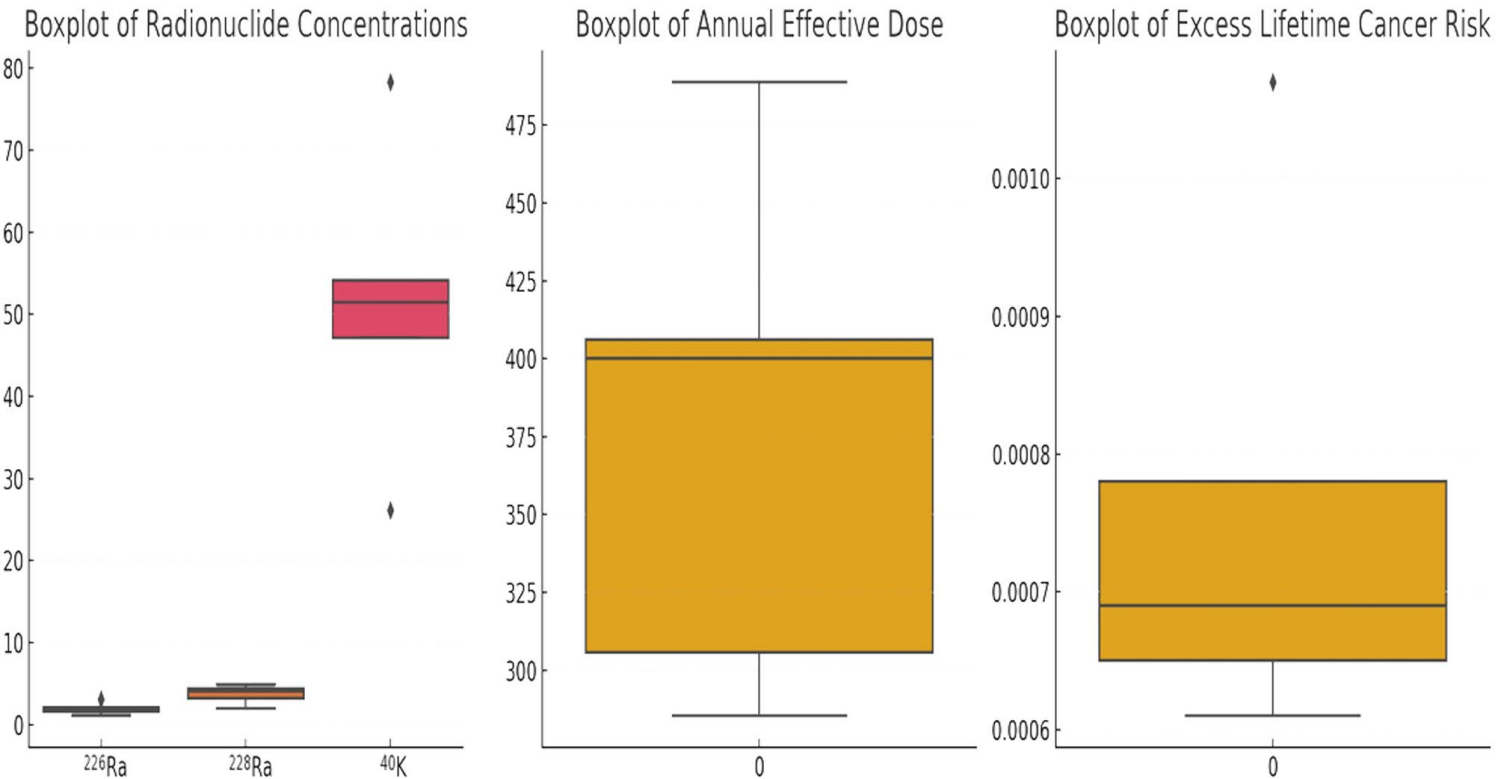
– Boxplots of highlighting distribution patterns.

Distribution patterns and potential outliers shows as form of boxplots in Fig. 3. Potassium shows wider variation among samples as potassium is naturally abundant in foodstuffs including rice Annual Effective Dose (AED) and Excess Lifetime Cancer Risk (ELCR) have consistent ranges without significant outliers, reflecting stability in radiological risk across the tested rice samples.

However, despite these encouraging results, caution must be exercised, particularly concerning vulnerable groups such as children and pregnant women as cumulative intakes of these natural radionuclide via rice consumption may contribute to incremental internal radiological risk over decades which may lead to some forms of bone and/or blood cancers. These populations might have increased susceptibility to radiation due to physiological and dietary differences. Hence, future research efforts should prioritize age-specific assessments to refine dietary guidelines and enhance public health interventions.

This research is subject to some specific limitations. First, while the sample size per brand conforms to international radiological food-assessment standards, it may not comprehensively represent the complete range of imported rice brands entering the Ghanaian market as sampling was restricted to key markets in Accra, Kumasi, and Tamale, and as a result, may not accurately represent nationwide distribution patterns or informal import channels. Furthermore, potential seasonal or interannual variability in radionuclide absorption within the countries of origin was not evaluated, given that the study design encompassed only a single-year sampling campaign. Future research incorporating larger sample sizes, broader geographic coverage, and multi-seasonal data collection would yield a more comprehensive assessment of radiological hazards.

From a policy and regulatory perspective, this research underscores the necessity for systematic monitoring and risk assessment mechanisms within Ghana’s food safety infrastructure. Strengthening domestic regulations to harmonize with international safety standards recommended by organizations such as IAEA, ICRP, and UNSCEAR would be beneficial. Such measures would not only boost public confidence in food safety but also guarantee compliance with international best practices, safeguarding public health.

## Conclusion

This study assessed radiological health risks associated with radionuclides in imported rice consumed in Ghana. This study identified measurable concentrations of radionuclides in imported rice samples, establishing a foundation for assessing potential public health risks. The ^226^Ra, ^228^Ra, and ^40^K activity concentrations were within globally accepted limits. The estimated annual effective dose remained below safety thresholds of 1 mSv/year, indicating negligible radiological health risks. The ELCR values indicate a below-world average but noteworthy potential health risk from long-term consumption of imported rice containing natural radionuclides.

While these findings indicate some level of radiological exposure through rice consumption, the risk remains minimal within the context of international safety guidelines. Given the country’s dependence on imported rice to meet dietary demands implementing a systematic surveillance and risk assessment protocols would be instrumental in ensuring food safety and public health protection while aligning Ghanaian radiological safety standards with international benchmarks set by organizations such as the IAEA, ICRP and UNSCEAR. This will go a long way to strengthening domestic regulations and harmonizing them with global safety frameworks and enhance public confidence.

Also, because of the potential for greater dose sensitivity in children and pregnant women, future studies should investigate age-specific dose evaluations. National risk evaluations might be improved with a broader sample that incorporates more imported brands, local variations, and various distribution routes. The case for conducting annual investigations into the fluctuation of radionuclide concentrations would be made even stronger with the help of longitudinal research, which would provide the groundwork for permanent regulating and monitoring systems.

## Data Availability

All data is contained in the manuscript.
